# Multiple lifestyle factors and depressed mood: a cross-sectional and longitudinal analysis of the UK Biobank (*N* = 84,860)

**DOI:** 10.1186/s12916-020-01813-5

**Published:** 2020-11-12

**Authors:** Jerome Sarris, Russell Thomson, Fiona Hargraves, Melissa Eaton, Michael de Manincor, Nicola Veronese, Marco Solmi, Brendon Stubbs, Alison R. Yung, Joseph Firth

**Affiliations:** 1 NICM Heath Research Institute, Western Sydney University, Westmead, NSW 2145 Australia; 2 Professorial Unit, The Melbourne Clinic, Department of Psychiatry, The University of Melbourne, Melbourne, VIC Australia; 3 Centre for Research in Mathematics and Data Science, Western Sydney University, Parramatta, NSW Australia; 4 National Research Council, Neuroscience Institute, Aging Branch, Padua, Italy; 5 Neurosciences Department, University of Padua, Padua, Italy; 6 Padua Neuroscience Center, University of Padua, Padua, Italy; 7 Physiotherapy Department, South London and Maudsley NHS Foundation Trust, Denmark Hills, London, SE5 8AZ UK; 8 Department of Psychological Medicine, Institute of Psychiatry, Psychology and Neuroscience, King’s College London, De Crespigny Park Box, London, SE5 8AF UK; 9 Orygen, Department of Psychiatry, Melbourne University, Melbourne, VIC Australia; 10 Division of Psychology and Mental Health, University of Manchester, Manchester, UK

**Keywords:** Lifestyle medicine, Mood disorders, Diet, Physical activity, Screen time, Health

## Abstract

**Background:**

There is now evolving data exploring the relationship between depression and various individual lifestyle factors such as diet, physical activity, sleep, alcohol intake, and tobacco smoking. While this data is compelling, there is a paucity of longitudinal research examining how multiple lifestyle factors relate to depressed mood, and how these relations may differ in individuals with major depressive disorder (MDD) and those without a depressive disorder, as ‘healthy controls’ (HC).

**Methods:**

To this end, we assessed the relationships between 6 key lifestyle factors (measured via self-report) and depressed mood (measured via a relevant item from the Patient Health Questionnaire) in individuals with a history of or current MDD and healthy controls (HCs). Cross-sectional analyses were performed in the UK Biobank baseline sample, and longitudinal analyses were conducted in those who completed the Mental Health Follow-up.

**Results:**

Cross-sectional analysis of 84,860 participants showed that in both MDD and HCs, physical activity, healthy diet, and optimal sleep duration were associated with less frequency of depressed mood (all *p* < 0.001; ORs 0.62 to 0.94), whereas screen time and also tobacco smoking were associated with higher frequency of depressed mood (both *p* < 0.0001; ORs 1.09 to 1.36). In the longitudinal analysis, the lifestyle factors which were protective of depressed mood in both MDD and HCs were optimal sleep duration (MDD OR = 1.10; *p* < 0.001, HC OR = 1.08; *p* < 0.001) and lower screen time (MDD OR = 0.71; *p* < 0.001, HC OR = 0.80; *p* < 0.001). There was also a significant interaction between healthy diet and MDD status (*p* = 0.024), while a better-quality diet was indicated to be protective of depressed mood in HCs (OR = 0.92; *p* = 0.045) but was not associated with depressed mood in the MDD sample. In a cross-sectional (OR = 0.91; *p* < 0.0001) analysis, higher frequency of alcohol consumption was surprisingly associated with reduced frequency of depressed mood in MDD, but not in HCs.

**Conclusions:**

Our data suggest that several lifestyle factors are associated with depressed mood, and in particular, it calls into consideration habits involving increased screen time and a poor sleep and dietary pattern as being partly implicated in the germination or exacerbation of depressed mood.

## Background

Despite advances in pharmacological and psychological treatment options for depression, the condition is still pervasive, having a population prevalence in Western societies of approximately 7% per year [1, 2]. Understanding potential modifiable risk factors is an essential first step to prevent the burden of depression. A recent umbrella review of non-genetic risk factors identified that six risk factors met the criteria for convincing evidence for being associated with depression, including widowhood, physical abuse during childhood, obesity, having four to five metabolic risk factors, sexual dysfunction, and job strain [3].

Recently, interest has grown in the potential relationship between lifestyle patterns and depression, in particular assessing whether people engaging in a healthy lifestyle may have a better mood compared to unhealthy counterparts. Key analyses to date tend to explore the association with physical activity [4] and also dietary quality [5], although the relationships with other modifiable lifestyle elements have been generally less explored. These lifestyle factors are all modifiable, thus providing an additional array of treatment options in concert with existing strategies. In fact, for milder forms of depression, notable treatment guidelines now recommend addressing lifestyle factors before commencing pharmacotherapy [6].

Healthy lifestyle elements are primarily considered to involve a healthy diet (higher in plant foods and lean protein, and lower in refined and ultra-processed foods); adequate (but not extreme) physical activity; adequate sleep duration and quality; avoidance or minimisation of alcohol, smoking, or substance use; and sufficient exposure to sunlight and nature [7]. Other factors which may be included within the lifestyle domain include relationship interactions (including with animals), hobbies, and time spent interfacing with technology, e.g. TV or Internet [8].

In respect to the potential neurobiological pathways through which various lifestyle factors impact mental health, data exists revealing an anti-inflammatory effect from both exercise [9] and diet [10–12]. Raised inflammation has a strong pathological link with depression. There is also data showing that smoking or poor sleep may also have pro-inflammatory effects in humans [13, 14]. Chronic low-grade inflammation has been linked with a broad range of psychiatric disorders [15]. An additional pathway modified by lifestyle factors concerns the role of the gut microbiome. The microbiome can be influenced by both physical activity [16] and diet [17], and a healthy microbiome may also potentially influence mood levels. Downstream consequences may also occur from poor health behaviours. For example, insufficient exercise, a poor diet, and insomnia can contribute factors towards the development of obesity, which in turn can raise inflammatory markers and have a negative effect on self-image and self-esteem [18]. Another simpler link can be found regarding nutrient-depleted diets not providing the vital micronutrients required for optimal neurological health [19].

In recent years, some research has assessed how a range of lifestyle factors may influence our mental wellbeing, with several cross-sectional studies, for example [20–23] revealing that other elements such as a healthy diet, adequate duration of sleep, outdoor or sunlight exposure, alcohol limitation, and smoking avoidance are beneficial behaviours which relate to general mental health and mood. This data is compelling and stands to reason due to the well-documented effects that nutrition, sleep, physical activity, sunlight and air quality, and harmful recreational substances (i.e. drugs and alcohol) have on the brain [7].

However, due to the cross-sectional design of the aforementioned studies, directionality could not yet be established. Few studies have examined the relationships of multiple lifestyle factors with future depressive symptoms. For instance, Adjibade and colleagues [24] assessed hazard ratios (estimated using Cox proportional hazards models) in addition to population attributable risks, for the mental health of a French sample of 25,837 adults, and examined the prospective relationship to five modifiable lifestyle elements (diet, smoking, alcohol consumption, weight, and physical activity). From these lifestyle factors, they created a healthy lifestyle index. After 5 years of follow-up, there were a total of 2112 cases of de novo (incident) depression, as assessed via a Centre for Epidemiological Studies Depression Scale [French version] score of ≥ 17 and 23 for men and women (range 0–60), respectively. After accounting for confounding factors, the data revealed a significant longitudinal link between an overall healthy lifestyle and fewer emergent cases of depression.

While there is a growing body of research investigating how lifestyle factors affect the onset of depressive disorders [25], the literature has yet to address how lifestyle factors may differentially affect mood state in the general population compared to those living with major depressive disorder. Additionally, more research is needed on the potential psychological impact of increasing screen time, which has raised concerns in recent years [26]. Our rationale for conducting this study was to address these current deficits in the field and to assess multiple lifestyle measures as they are commonly interrelated. As detailed above, there is currently only limited evidence exploring the relationship between lifestyle factors and depressed mood.

To address these current deficits in the field, we conducted both a cross-sectional and a longitudinal analysis of the UK Biobank data to assess the relationship of key lifestyle factors (objectively measured physical activity, diet quality, adequate sleep, sedentary screen time, smoking status, and alcohol intake frequency) with frequency of depressed mood in both people with major depression and those without depression. Our a priori hypothesis was that healthy lifestyle behaviours would be associated with less depressed mood symptoms cross-sectionally, while being found to be protective against depressed mood across time in a longitudinal analysis. This was undertaken as it was vital to assess lifestyle behaviours in both cohorts to contrast any differences and also to determine if a baseline depressive disorders would prospectively affect the level of depressed mood.

## Methods

Generic ethical approval was granted for the UK Biobank from the NHS Research Ethics Committee (Ref. 11/NW/0382). The analyses presented in this study were provided separate approval by the UK Biobank research committee and were performed on data collected between 2007 and 2010 for baseline and 2016 and 2017 for longitudinal analyses. In brief, the UK Biobank is a nationwide, health-oriented, cohort study which takes place across 22 assessment centres throughout the UK [27]. The national Biobank collects data across the UK in order to investigate how various lifestyle, environmental, and genetic factors are associated with a range of health outcomes.

Potential participants were accessed via mail (sent to around 9.2 million homes in the UK). From this, a total of 502,664 participants were recruited, aged 37–73, and were required to attend UK Biobank assessment centres for extensive data collection, which included touchscreen questionnaires, in-person interviews, and physical health examinations [27]. Further details on the aims, methods, and procedures for the UK Biobank study are available elsewhere [27].

### Participant sampling

Participants used for this study were all those from the UK Biobank who could be categorised into groups of people who presented with previous or current major recurrent depression (MDD sample) and those without depressive disorders as healthy controls (HC) (i.e. no indication of present or previous affective disorders). This categorisation was based on a previous study, which assessed the prevalence of mood disorders within the UK Biobank cohort [28]. The study applied a pre-established criterion to individuals’ answers to the Structured Clinical Interview for DSM-IV Axis I Disorders (SCID-1) and Patient Health Questionnaire (PHQ) [28] to categorise participants as likely having a single episode of major depression, recurrent major depression, bipolar disorder, or no indicated mood disorders. The data revealed that lifetime prevalence rates were consistent with general population-based estimates for a current unipolar depressive episode across both genders [1]. For our study, the MDD group consisted of all those falling into the categories of recurrent major depression as described above at baseline, and any individuals with a previous/current ICD-10 diagnosis of recurrent major depression on UK Biobank hospital records. The HCs were those with no historical or current depressive disorder indicated from these measures at baseline. For our study, participants with neuropsychiatric conditions known to affect cognitive functioning and severe mental illnesses (such as schizophrenia other psychotic disorders and bipolar disorder) were excluded from the analyses. The supplementary material displays the excluded neurological conditions and their UK Biobank field codes.

### Lifestyle factor assessment

We chose to analyse the most pertinent lifestyle-related variables we could find available on the UK Biobank database. The lifestyle factors included physical activity, diet, sleep, smoking, screen time (greater use may pertain to a more sedentary lifestyle), and alcohol frequency all of which have individually been implicated in depression [8]. All of these were assessed using computerised questionnaires administered to participants at the baseline visit to UK Biobank assessment centres. The details on each of these factors and their respective UK Biobank ‘Field Codes/Categories’ (FCs) identifiers are given below:

#### Physical activity

The self-reported physical activity data available in the UK Biobank was used for this study, as only self-report physical activity was recorded immediately at baseline (while objective measures were collected later). The self-reported physical activity data were converted to metabolic equivalent of task (MET) minutes per week, using the data produced by a previous study (ref: https://bmjopen.bmj.com/content/6/3/e010038) now available within the UK Biobank (FC: 22040).

#### Diet

This was based on the food frequency questionnaire (FC: 100052). To determine individuals classified as having a ‘healthy diet’ for our analysis, the data on self-reported weekly food intake was used to determine those with (i) high intake of fruit and vegetables, (ii) high intake of fish, and (iii) low intake of processed meats and red meats. Those individuals who fulfilled at least two of those three criteria above were defined as having a ‘healthy diet’ (categorical variable). Further details are provided in the supplementary material for the diet categorisation used.

#### Sleep

Asking ‘About how many hours of sleep do you get in every 24 h? (please include naps)’ (FC: 1160), participants who achieved 7 to 9 h were classified as ‘optimal sleep’. This is based off Australian Sleep Foundation Guidelines for adults (however, it is recognised that in less active older adults that 7 to 8 h of sleep per night may be more optimal) (https://www.sleephealthfoundation.org.au/files/pdfs/Sleep-Needs-Across-Lifespan.pdf).

#### Smoking

Using the question ‘Do you smoke tobacco now?’ (FC: 1239), for the purposes of this study, participants were classified as either ‘non-smokers’ (never or not current smokers) or ‘smokers’ (still current smoking).

#### Sedentary screen time

Sedentary screen time is calculated in hours per week, adding together the participants’ answers to the questions ‘In a typical DAY, how many hours do you spend watching TV?’ (FC: 1070) and ‘In a typical DAY, how many hours do you spend using a computer? (not including time using a computer at work)’ (FC: 1080).

#### Alcohol frequency

Alcohol frequency is categorised as 6 = ‘daily’, 5 = ‘three to four times per week’, 4 = ‘once or twice per week’, 3 = ‘one to three times per month’, 2 = ‘special occasions only’, or 1 = ‘never’ using participant response to the question ‘About how often do you drink alcohol?’ (FC: 1558). For the purposes of this study, this ordinal variable was treated as linear, so that results can be interpreted as a dose-response relationship.

#### Depressive mood assessment

Frequency of depressed mood were assessed over the last 2 weeks, with the following question of a computerised questionnaire administered at the baseline visit:

‘ Over the past two weeks, how often have you felt down, depressed or hopeless?’ (FC: 2050).

And this similarly worded question, assessing the same construct of frequency of depressed mood, at follow-up (administered via an online survey):

‘Over the last 2 weeks, how often have you been bothered by any of the following problems? Feeling down, depressed, or hopeless’ (FC: 20510).

With participants responding as ‘Prefer not to Answer’, ‘Not at all’, ‘Several days’, ‘More than half of days’, and ‘Nearly every day’ in both cases.

The answers were assigned scores of missing for ‘prefer not to answer’ then 1, 2, 3, or 4, respectively, for frequency of depressed mood, and used as an ordinal measure.

### Confounding variables

Demographic information was collected from computerised questionnaires completed at UK Biobank assessment centres. Age, gender, ethnicity, social deprivation, education, and BMI were identified as the key confounding variables. Highest education/qualifications responses were dichotomized to categorise participants as either having or not having university/college degree-level qualifications. As ~ 95% of the sample were classified as Caucasian, ethnicity was categorised as ‘Caucasian’ or ‘Other’. Social deprivation was assessed using the Townsend deprivation index (using participants’ postcodes on joining the study). BMI measurement was acquired on-site via a research assistant who conducted the physical health assessments.

### Statistical analysis

Ordinal logistic regression was used to examine the association between lifestyle factors and frequency of depressive moods in the last 2 weeks throughout this manuscript. The frequency of depressive mood variable was ordinal in nature, with four possible levels (1 = ‘Not at all’, 2 = ‘Several days’, 3 = ‘More than half of days’, and 4 = ‘Nearly every day’). The cross-sectional analysis used the frequency of depressive moods at the same time that the lifestyle factors were measured as the outcome variable. Lifestyle factors included physical activity (MET minutes), diet, optimal hours of sleep per night (7 to 9 h), current smoking, screen time, and alcohol frequency. The confounders for which we accounted were ethnicity, social deprivation, gender, age, and optimal BMI (18.5–25).

An ordinal logistic regression model was also fitted with the outcome variable: frequency of depressive moods at follow-up from those participants taking part in the UK Biobank study (collected from 2016 to 2017). Lifestyle factors at baseline and confounders were the predictors in this model. As the frequency of depressive moods at baseline was included as a covariate in this model, effect sizes represented change in the frequency of depressive mood from baseline to follow-up.

To assess if the lifestyle factors affect the frequency of depressive moods differently between those participants with/without a depressive disorder (MDD vs HCs), separate models were fitted for these two groups. A model that included interactions between the MDD status and each of the lifestyle factors was used to test whether they affected the frequency of depressive mood differently between those with MDD and those without. This approach was important as it was theorised that those with or without pre-existing depression may have a different trajectory of mood symptoms modulated by lifestyle factors.

For each of the models run, we reported appropriate summary statistics regarding the sample composition (sample size, averages and error surrounding measurements of interest), the model parameter estimates and associated confidence. The effect size of the lifestyle factors and confounders on the frequency of depressive mood were plotted using odds ratios and 95% confidence intervals. Wald’s test was employed for all *p* values and confidence intervals. Statistical analyses were conducted using R, with the help of the R function *polr* in the library, *MASS*. A *p* value of ≤ 0.05 was considered as statistically significant.

## Results

### Sample characteristics

The entire sample for cross-sectional analyses consisted of all 84,860 participants of the UK Biobank who (i) had provided sufficient information to establish MDD vs non-depression status, (ii) provided sufficient data on exposure and outcome measures to be included in the analyses and (iii) did not meet the exclusion criteria (as described above). The average age of the baseline sample was 55 for the MDD (*n* = 18,793) compared to 57 for the HCs (*n* = 66,067). A summary of the two groups is presented in Table 1. The only primary difference between groups was a greater number of females (65%) compared to males (35%) in the sub-sample with depression. The predominance of the sample identified as being of Caucasian ethnicity (94% and 91%, respectively). There were also significantly more smokers in the MDD group (13%) compared to the HCs (8%). Finally, there was a similar BMI level for both groups.

**Table 1:** Number of participants and their characteristics. Midrange BMI is defined as 18.5–25. Optimal sleep is defined as 7–9 h a night. Healthy diet is defined as having 2 or more of 3 healthy diet attributes (sufficiently low meat, high fruit/vegetable, high fish). Physical activity is measured in MET minutes per week. Screen time is measured as the number of hours of non-work computer and TV time per day. Alcohol frequency is given as 1 = ‘never’, 2 = ‘special occasions only’, 3 = ‘one to three times per month’, 4 = ‘once or twice per week’, and 5 = ‘three to four times per week’. Social deprivation measured using the Townsend deprivation index based on postcode

	Baseline measures		Baseline and follow-up measures	
	Depression	Non-depression	Depression	Non-depression
**Number of participants**	18,793	66,067	7050	24,293
**Age (mean (sd))**	55.3 (8)	57.1 (8.1)	55.1 (7.7)	56.8 (7.8)
**Women (%)**	64.6%	47.7%	67.2%	49.6%
**Caucasian (%)**	94.4%	92.1%	96.4%	95.5%
**Current smoker (%)**	12.8%	7.8%	9.1%	6.1%
**Midrange BMI (%)**	32%	34.3%	36.2%	39.3%
**Optimal sleep (%)**	70.5%	76.3%	74.1%	78.8%
**Healthy diet (%)**	38.3%	37%	39%	37.4%
**Physical activity (mean (sd))**	2663 (2743)	2765 (2712)	2493 (2488)	2588 (2458)
**Screen time (mean (sd))**	3.9 (2.4)	3.8 (2.2)	3.8 (2.3)	3.7 (2.1)
**Alcohol frequency (mean (sd))**	4 (1.6)	4.2 (1.5)	4.1 (1.5)	4.4 (1.4)
**Social deprivation (mean (sd))**	− 1 (3)	− 1.4 (2.8)	− 1.2 (2.8)	− 1.7 (2.6)

### Lifestyle factors affecting mood (cross-sectional data)

**Fig. 1:**
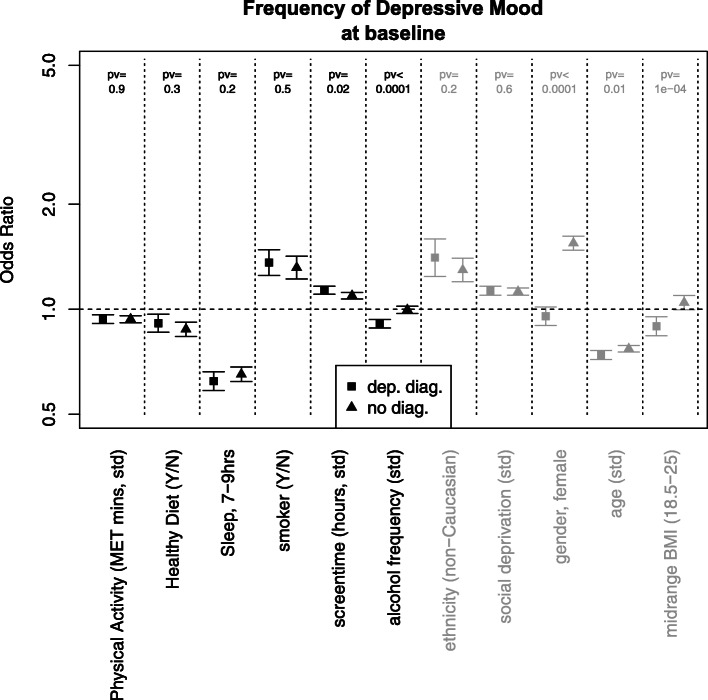
The odds ratios (and 95% confidence intervals) for an increased frequency of depressive mood at baseline, for a number of lifestyle factors and confounders (in grey) measured at baseline. Results are presented both for the samples with major depressive disorder (squares) and those without a depressive disorder (triangles). Odds ratios above 1 indicate that an increase in the given lifestyle factor is associated with more depressive moods at a similar time point. For the categorical variables (sleep, 7–9 h; smoker; ethnicity (non-Caucasian); gender (female); and midrange BMI), the reference group is all other participants. *std* = standardised variables. *pv* = *p*-values for interaction

Increased physical activity, as measured by a one sd increase in MET minutes per week (MDD group: OR = 0.94, 95% CI 0.91–0.96, *p* < 0.0001; HC group: OR = 0.94, 95% CI 0.92–0.96, *p* < 0.0001), healthy diet (MDD group: OR = 0.91, 95% CI 0.86–0.97, *p* = 0.0026; HCs: OR = 0.88, 95% CI 0.84–0.92, *p* < 0.0001) and optimal sleep (MDD group: OR = 0.62, 95% CI 0.58–0.66, *p* < 0.0001; HCs: OR = 0.65, 95% CI 0.62–0.68, *p* < 0.0001) were significantly cross-sectionally associated with less depressive mood in both groups (Fig. 1, Online Table 1).

Conversely, being a current smoker (MDD group: OR = 1.36, 95% CI 1.25–1.48, *p* < 0.0001; HCs: OR = 1.32, 95% CI 1.22–1.42, *p* < 0.0001) and more screen time (one sd increase) (MDD group: OR = 1.13, 95% CI 1.1–1.16, *p* < 0.0001; HCs: OR = 1.09, 95% CI 1.07–1.12, *p* < 0.0001) were significantly associated with increased depressive mood in both groups.

Highly significant interactions were observed between a MDD status and alcohol frequency (all *p* values ≤ 0.0001). Alcohol frequency had a stronger negative cross-sectional (OR = 0.91, 95% CI 0.88–0.94, *p* < 0.0001) association with the frequency of depressive mood in those with MDD compared with those without depression. Cross-sectional associations with confounding variables are displayed in Online Table 1.

### Lifestyle factors affecting mood—longitudinal data

**Fig. 2:**
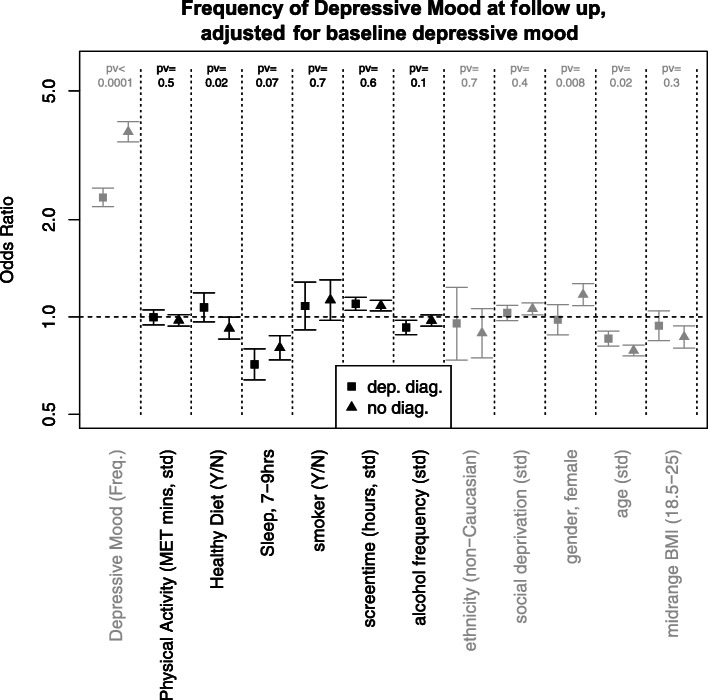
The odds ratios (and 95% confidence intervals) for an increased frequency of depressive mood at baseline and at follow-up, for a number of lifestyle factors and confounders (in grey) measured at baseline. The variable labelled ‘Depressive Mood (Freq.)’ is measured at baseline. Results are presented both for the samples with major depressive disorder (squares) and those without a depressive disorder (triangles). Odds ratios above 1 indicate that an increase in the given lifestyle factor is associated with an increase in depressive moods from baseline to follow-up. For the categorical variables (sleep, 7–9 h; smoker; ethnicity (non-Caucasian); gender (female); and midrange BMI), the reference group is all other participants. *std* = standardised variables. *pv* = p-values for interaction

In respect to the longitudinal data, the lifestyle factors screen time (MDD group: OR = 1.1, 95% CI 1.05–1.15, *p* = 0.0001; HCs: OR = 1.08, 95% CI 1.04–1.12, *p* < 0.0001) and optimal sleep (MDD group: OR = 0.71, 95% CI 0.64–0.8, *p* < 0.0001; HCs: OR = 0.80, 95% CI 0.74–0.87, *p* < 0.0001) were prospectively associated with depressed mood at follow-up for both the MDD group and HCs. These models adjusted for baseline depressed mood, indicating that more screen time and non-optimal sleep were associated with an increase in the frequency of depressed mood from baseline to follow-up (Fig. 2).

There was also a significant interaction between healthy diet and MDD status (*p* = 0.024). A healthy diet at baseline was significantly associated with a decrease in the frequency of depressed moods at follow-up in the HCs (OR = 0.92, 95% CI 0.85–0.998, *p* = 0.045) but not in the MDD group (OR = 1.07, 95% CI 0.97–1.19, *p* = 0.20). Longitudinal associations with confounding factors are displayed in Online Table 2.

## Discussion

The data from one of the largest analyses of lifestyle data and mental health showed, as expected, that a range of elements were associated with increased depressive moods in the cross-sectional analyses. Results provided a strong indication that optimal sleep with less screen time may protect against depressive symptoms over time, in both those with depressive disorders and those without. Having a healthy diet was associated with reduced frequency of depressed mood at follow-up in HCs, but not those with pre-existing depressive disorders. Other factors, such as being a current smoker and physical activity levels, were not prospectively associated with depressed mood at follow-up. A likely reason the physical activity (PA) variable did not align with our hypothesis regarding the potential protective effect of lifestyle behaviours on depressed mood concerns the discrepancy between self-reported and ‘actual’ PA [29]. Indeed, previous research in the UK Biobank has found objectively measured physical activity is protective of depression onset, while self-reported levels of physical activity are not [30], and the same may apply for depressive moods. However, due to the objective physical activity data within the UK Biobank cohort being collected several years after baseline assessments, only the self-report measures were suitable for our analyses. Another curious finding concerned the observation that a higher frequency of alcohol consumption was associated with lower frequency of depressive moods among those with MDD. While the damage of heavy alcohol use has been well-established, it is possible that people with depression may use alcohol as a form of self-medication, which for some may reduce the subjective experience of depressed mood being replaced by effects of alcohol. Overall, the effect size was small and neglectable compared with other detrimental effects of alcohol.

The cross-sectional findings are in concert with the majority of research, with the longitudinal data in part aligning with Adjibade and colleagues [24] who found a prospective relationship to five lifestyle/health elements (smoking, regular physical activity, diet quality, alcohol consumption and BMI). Their formulated healthy lifestyle index revealed a significant prospective link between overall healthy lifestyle and fewer de novo cases of depression. We do acknowledge that the use of a form of healthy lifestyle index may however be a helpful approach, as it is important to capture if possible a potential cumulative effect of healthy lifestyle behaviours on reducing depressive symptoms. To date, however, such indexes have not been validated, and further work is needed before adopting this as a standard approach. As recent work has found that many lifestyle and social factors which appear to contribute towards depression onset in prospective analyses may not hold causal relations [25], further research is also required to determine the underpinning causality of lifestyle factors with regards to depressive moods in both those with MDD and the general population.

Our results may inform public health policy by further highlighting the important relationship between people being encouraged and supported to engage in a range of health-promoting activities, in particular maintaining optimal sleep and lessening screen time, may reduce the frequency of depressive moods in those with and without depressive disorders. It is interesting to note that those without depression who had a healthier diet at baseline had a significantly lower risk of depressive symptoms at follow-up, while this effect was not evident in those with MDD at baseline. One explanation for this could potentially be due to those with depression having a pre-existent poorer dietary pattern, which was maintained over time. In respect to the relationship between higher screen time with increased frequency of depressed mood, it has yet to be established if these relations are due to the sedentary nature of screen time activities or due to the potential ‘direct’ psychological impact of content absorbed from TV time/computer time itself. In either case, this may also be coupled as an exacerbatory factor with the established link between poor sleep and increased risk of depression [31], as high levels of screen time may displace sleep time or otherwise negatively impact on sleep quality itself.

In respect to interventional approaches, there is solid data in terms of prescriptive exercise to improve mood, while there is less so for other modifiable lifestyle elements. The evidence underpinning dietary programmes as reducing depressive symptoms is also evolving [32], and a number of recent RCTs have further found that Mediterranean diet interventions have moderately large effects on symptoms of depression in those with clinical depressive disorders [33–35]. Other meta-analyses of non-pharmacological sleep enhancement programmes have also found evidence for reductions in depressive symptoms [36]. Reduction of harmful behaviours such as smoking or excessive alcohol misuse has also been shown to improve mood [37]. What is less known, however, is the impact of actively reducing screen time, interaction with excessive social media and multi-tasking interface with technology [38, 39].

From a clinical perspective, lifestyle programmes need to be supportive, personalised and ideally consist of a multi-component approach, especially to address physical comorbidities which often co-exist with depression. While the findings in our paper do not support the relationship between all lifestyle factors modifying depressed mood over time, the combination of the cross-sectional and longitudinal data, considered alongside the findings of other recent studies, does support the use of multi-pronged lifestyle intervention as being an effective approach (as embryonic RCT data has revealed [40]). An example of a multi-pronged lifestyle intervention is shown in the recently published NEWTx pilot study, which examined the intervention of nutrition, exercise and wellness via general education modules in 38 people with bipolar disorder (19 vs 19; active programme vs waitlist; assessors were blinded) [41]. While the treatment group reported a range of beneficial effects on their wellbeing and symptoms, only improvements in general functioning were significantly greater in the NEWTx group than in the control group. There were no group differences in weight loss or mood symptoms over the study duration (although the treatment group did have a trend for a reduction of no over time). The authors commented that a manualised generalised approach does not appear to be sufficient, and a more personalised approach according to their needs (e.g. physical capability, medications, diagnoses) is needed to achieve a greater effect. This is in keeping with the recommendations of the recent Lancet Psychiatry Commission regarding the delivery of multi-component lifestyle interventions for people with mental illness [42].

While our paper reflects several strengths, including both the cross-sectional and longitudinal analysis of the largest cohort with lifestyle and mood data to date, limitations are recognised for this analysis. Principally, it is acknowledged that most of the lifestyle data collected via the UK Biobank were based on self-report measures, which have been shown to produce inaccurate representations of lifestyle risk factors [29] It is also understood that the UK Biobank is not a perfect socioeconomic representation of UK residents and that selection bias into the study occurred (due in part to a potential over-representation of an older more educated Caucasian population). Analyses were conducted using listwise deletion, and as such, the results represent the sample where all data was available. A further limitation is that depressed mood was not assessed via a specialised clinician-rated depression scale (such as the Hamilton Depression Rating Scale), and instead based on a single-item measure of the recent frequency of depressed mood. The strengths however include the large sample, separation of participants into MDD and HC at baseline, and prospective nature of the analyses.

## Conclusions

We conclude from our analysis of the UK Biobank lifestyle and mood data that over all, a range of lifestyle factors are related to depression and a range of lifestyle factors were found to potentially provide a significant protective factor for the frequency of depressive mood at longitudinal follow-up. Future research should look at more complex modelling to assess the potential of various lifestyle elements to influence mood. The design of interventions should also consider more integrated approaches. This is best enacted via supported individualised, tailored formats which are adaptable to participants’ needs and capabilities.

## Supplementary information

**Additional file 1: Table S1.** Results of the cross-sectional analysis on the association between frequency of depressive moods and lifestyle factors and confounders. **Table S2.** Results of the longitudinal analysis on the association between frequency of depressive moods at follow-up and lifestyle factors and confounders. **Table S3.** Excluded neuropsychiatric disorders from UK Biobank records.

## Data Availability

The data used in the analyses contained within the publication are available via the UK Biobank pending their consent for access.
