# Supplementary material for: Multiple lifestyle factors and depressed mood: a cross-sectional and longitudinal analysis of the UK Biobank (N = 84,860)
Source: BMC Med. 2020 Nov 12;18:354. doi: 10.1186/s12916-020-01813-5 (PMC7661271; doi:10.1186/s12916-020-01813-5)
Supplement: Supplementary file 1 — Additional file 1: Table S1. Results of the cross-sectional analysis on the association between frequency of depressive moods and lifestyle factors and confounders. Table S2. Results of the longitudinal analysis on the association between frequency of depressive moods at follow-up and lifestyle factors and confounders. Table S3. Excluded neuropsychiatric disorders from UK Biobank records. [file 12916_2020_1813_MOESM1_ESM.docx]

**Supplementary information**

|  | **Beta Value** | **Std. Error** | **OR** | **Lower 95% CI** | **Upper 95% CI** | **p value** |
| --- | --- | --- | --- | --- | --- | --- |
| **Physical Activity (MET minutes, std)** | -0.0649 | 0.0119 | 0.937 | 0.916 | 0.959 | < 0.0001 |
| **depression diagnosis** | 2.19 | 0.18 | 8.94 | 6.29 | 12.7 | < 0.0001 |
| **Diet (std)** | -0.132 | 0.0239 | 0.877 | 0.836 | 0.919 | < 0.0001 |
| **Sleep, 7-9hrs** | -0.428 | 0.0246 | 0.652 | 0.621 | 0.684 | < 0.0001 |
| **smoker** | 0.273 | 0.0382 | 1.31 | 1.22 | 1.42 | < 0.0001 |
| **screentime (std)** | 0.0889 | 0.011 | 1.09 | 1.07 | 1.12 | < 0.0001 |
| **alcohol (Freq., std)** | -0.00412 | 0.012 | 0.996 | 0.973 | 1.02 | 0.73 |
| **ethnicity** | 0.256 | 0.0395 | 1.29 | 1.2 | 1.4 | < 0.0001 |
| **social dep (std)** | 0.116 | 0.0123 | 1.12 | 1.1 | 1.15 | < 0.0001 |
| **female** | 0.435 | 0.0235 | 1.54 | 1.48 | 1.62 | < 0.0001 |
| **age (std)** | -0.26 | 0.0111 | 0.771 | 0.754 | 0.788 | < 0.0001 |
| **midrange BMI (18.5-25)** | 0.0441 | 0.024 | 1.05 | 0.997 | 1.1 | 0.066 |
| **Physical Activity * depression diagnosis** | -0.00191 | 0.0189 | 0.998 | 0.962 | 1.04 | 0.92 |
| **Diet * depression diagnosis** | 0.0378 | 0.039 | 1.04 | 0.962 | 1.12 | 0.33 |
| **Optimal sleep * depression diagnosis** | -0.0578 | 0.0403 | 0.944 | 0.872 | 1.02 | 0.15 |
| **smoker* depression diagnosis** | 0.0434 | 0.0581 | 1.04 | 0.936 | 1.17 | 0.46 |
| **Screentime * depression diagnosis** | 0.0404 | 0.0176 | 1.04 | 1.01 | 1.08 | 0.022 |
| **Alcohol frequency * depression diagnosis** | -0.0934 | 0.019 | 0.911 | 0.877 | 0.945 | < 0.0001 |
| **ethnicity* depression diagnosis** | 0.0977 | 0.075 | 1.1 | 0.952 | 1.28 | 0.1927 |
| **Social deprivation * depression diagnosis** | 0.00932 | 0.0199 | 1.01 | 0.971 | 1.05 | 0.6388 |
| **Female * depression diagnosis** | -0.482 | 0.0393 | 0.617 | 0.571 | 0.667 | < 0.0001 |
| **Age * depression diagnosis** | -0.0479 | 0.0188 | 0.953 | 0.919 | 0.989 | 0.011 |
| **midrange BMI * depression diagnosis** | -0.158 | 0.0403 | 0.854 | 0.789 | 0.924 | 0.0001 |
| **1\|2** | 0.164 | 0.106 |  |  |  | 0.122 |
| **2\|3** | 2.23 | 0.108 |  |  |  | < 0.0001 |
| **3\|4** | 3.35 | 0.11 |  |  |  | < 0.0001 |

**Online Table 1:** Results of the cross-sectional analysis on the association between frequency of depressive moods and lifestyle factors and confounders, using ordinal regression, including interactions between depression diagnosis and the lifestyle factors and confounders. The rows 1|2, 2|3 and 3|4 indicates the relationship between the levels of the frequency of depressive moods (1=“Not at all”, 2=“Several days”, 3=“More than half of days”, 4=“Nearly every day”) under the proportional odds assumption. *Std* = standardised variables. For the categorical variables (sleep, 7-9hrs; smoker; ethnicity (non-Caucasian); gender (female) and midrange BMI), reference group is all other participants.

|  | **Beta Value** | **Std. Error** | **OR** | **Lower**  **95% CI** | **Upper**  **95% CI** | **p value** |
| --- | --- | --- | --- | --- | --- | --- |
| **Frequency of depressive mood at baseline** | 1.3 | 0.0364 | 3.66 | 3.41 | 3.93 | < 0.0001 |
| **depression diagnosis** | 0.996 | 0.339 | 2.71 | 1.39 | 5.27 | 0.003 |
| **Physical Activity (MET minutes, std)** | -0.0242 | 0.0212 | 0.976 | 0.936 | 1.02 | 0.25 |
| **Diet (std)** | -0.0792 | 0.04 | 0.924 | 0.854 | 0.999 | 0.048 |
| **Sleep, 7-9hrs** | -0.219 | 0.0439 | 0.804 | 0.738 | 0.876 | < 0.0001 |
| **smoker** | 0.12 | 0.0735 | 1.13 | 0.975 | 1.3 | 0.10 |
| **screentime (std)** | 0.0794 | 0.019 | 1.08 | 1.04 | 1.12 | < 0.0001 |
| **alcohol (Freq., std)** | -0.0252 | 0.0207 | 0.975 | 0.937 | 1.02 | 0.22 |
| **ethnicity** | -0.115 | 0.089 | 0.891 | 0.747 | 1.06 | 0.20 |
| **social dep (std)** | 0.057 | 0.0219 | 1.06 | 1.01 | 1.11 | 0.009 |
| **female** | 0.159 | 0.0396 | 1.17 | 1.09 | 1.27 | 1e-04 |
| **age (std)** | -0.238 | 0.0196 | 0.788 | 0.758 | 0.819 | < 0.0001 |
| **midrange BMI (18.5-25)** | -0.141 | 0.0401 | 0.869 | 0.803 | 0.939 | 4e-04 |
| **Frequency of depressive mood at baseline * depression diagnosis** | -0.408 | 0.0495 | 0.665 | 0.603 | 0.732 | < 0.0001 |
| **Physical Activity * depression diagnosis** | 0.0217 | 0.0349 | 1.02 | 0.954 | 1.09 | 0.53 |
| **Diet * depression diagnosis** | 0.15 | 0.0665 | 1.16 | 1.02 | 1.32 | 0.024 |
| **Optimal sleep * depression diagnosis** | -0.132 | 0.0718 | 0.876 | 0.761 | 1.01 | 0.066 |
| **smoker* depression diagnosis** | -0.0384 | 0.115 | 0.962 | 0.769 | 1.2 | 0.73 |
| **Screentime * depression diagnosis** | 0.0182 | 0.0306 | 1.02 | 0.959 | 1.08 | 0.55 |
| **Alcohol frequency * depression diagnosis** | -0.053 | 0.0334 | 0.948 | 0.888 | 1.01 | 0.113 |
| **ethnicity* depression diagnosis** | 0.0698 | 0.161 | 1.07 | 0.782 | 1.47 | 0.66 |
| **Social deprivation * depression diagnosis** | -0.0271 | 0.0357 | 0.973 | 0.908 | 1.04 | 0.45 |
| **Female * depression diagnosis** | -0.182 | 0.0682 | 0.834 | 0.73 | 0.953 | 0.007 |
| **Age * depression diagnosis** | 0.0795 | 0.034 | 1.08 | 1.01 | 1.16 | 0.019 |
| **midrange BMI * depression diagnosis** | 0.0766 | 0.0678 | 1.08 | 0.945 | 1.23 | 0.26 |
| **1\|2** | 1.41 | 0.198 |  |  |  | < 0.0001 |
| **2\|3** | 3.76 | 0.201 |  |  |  | < 0.0001 |
| **3\|4** | 4.8 | 0.205 |  |  |  | < 0.0001 |

**Online Table 2:** Results of the longitudinal analysis on the association between frequency of depressive moods at follow-up and lifestyle factors and confounders, using ordinal regression, including interactions between depression diagnosis and the lifestyle factors and confounders. The rows 1|2, 2|3 and 3|4 indicates the relationship between the levels of the frequency of depressive moods (1=“Not at all”, 2=“Several days”, 3=“More than half of days”, 4=“Nearly every day”) under the proportional odds assumption. *std* = standardised variables. For the categorical variables (sleep, 7-9hrs; smoker; ethnicity (non-Caucasian); gender (female) and midrange BMI), reference group is all other participants.

| Field Codes for Conditions | **Field 6150** = 3  **Field 20001** = 1031 OR 1032  **Field 20002** = 1082 1083 1086 1524 1262 1397 1683 1245 1246 1491 1425 1433 1258 1263 1264 1266 1244 1583 1659 1259 1240 1434  **Fields 41202 and 41204 =** F20 – F29, F31 |
| --- | --- |
| Names of conditions | Bipolar affective disorder  Brain cancer/primary malignant tumour  Brain haemorrhage  Brain/intracranial abscess  Cerebral aneurysm  Cerebral palsy  Chronic/degenerative neurological problem  Dementia/Alzheimer's disease/cognitive impairment  Encephalitis  Epilepsy  Head injury  Infection of nervous system  Ischaemic stroke  Meningeal cancer/malignant meningioma  Meningioma (benign)  Meningitis  Motor neurone disease  Multiple sclerosis  Neurological injury/trauma  Neuroma (benign)  Other demyelinating condition  Other neurological problem  Parkinson's disease  Psychotic disorders (ICD-10 F20-F29)  Spina bifida  Stroke  Subarachnoid haemorrhage  Subdural haematoma  Transient ischaemic attack |

**Online Table 3:** Excluded neuropsychiatric disorders from UK Biobank records
